# Survival from non-Hodgkin lymphoma in England and Wales up to 2001

**DOI:** 10.1038/sj.bjc.6604605

**Published:** 2008-09-23

**Authors:** B Rachet, E Mitry, A Shah, N Cooper, M P Coleman

**Affiliations:** 1 Cancer Research UK Cancer Survival Group, Non-Communicable Disease Epidemiology Unit, Department of Epidemiology and Population Health, London School of Hygiene and Tropical Medicine, Keppel Street, London WC1E 7HT, UK; 2 Département d'Hépatogastroentérologie et Oncologie Digestive, Centre Hospitalo-Universitaire Ambroise-Paré, 9 avenue Charles de Gaulle, Boulogne F-92100, France; 3 Social and Health Analysis and Reporting Division, Office for National Statistics (Room FG/114), 1 Myddelton Street, London EC1R 1UW, UK

Non-Hodgkin lymphoma comprises a disparate set of malignancies of lymphoid tissue, other than Hodgkin's disease, with a wide variety of biological, clinical and prognostic features. The clinical classification of lymphomas has changed considerably in response to advances in medical knowledge, but it has been difficult to assimilate these changes into the International Classification of Diseases (ICD) for Oncology, used by population-based cancer registries for coding cases for international comparison of cancer incidence (Percy *et al*, 1984). The tenth revision of the ICD provided more rubrics for non-Hodgkin lymphoma (C82–C85, C91.4, C96) than the two available in ICD-9 (200, 202), but cancer registry data were only coded to ICD-10 from 1995 or later.

As a result, it is not yet possible to provide long-term comparisons of incidence and survival trends for specific types of non-Hodgkin lymphoma with population-based data. This is also true of mortality data, which were only coded to ICD-10 from 2000 or later, and which are derived from the limited diagnostic information on death certificates, as opposed to the medical records used to register newly diagnosed cases. Mortality data are generally reported for all the non-Hodgkin lymphomas combined. We present survival trends for the same broad category of all the non-Hodgkin lymphomas combined.

Non-Hodgkin lymphoma is the most common lympho-haemopoietic malignancy in adults. It ranks as the eighth most common malignancy with almost 8000 new cases diagnosed each year. The male–female ratio is approximately 1.5. Non-Hodgkin lymphoma has tripled in frequency in both sexes since the early 1970s (Quinn *et al*, 2001), an increase echoed in many countries (Coleman *et al*, 1993). More than 60% of cases arise in persons aged 60 years or more, and the increase in incidence has been up to five-fold in older men and women, with little change in young adults. Incidence is slightly higher in affluent than deprived men, but there is no difference for women.

The causes of NHL are not well established, although solvents, pesticides and other chemicals have been implicated (Blair and Kazerouni, 1997; Lynge *et al*, 1997; Stellman, 1998). Certain viruses, such as Epstein–Barr virus, HIV and HTLV are known to be potentially lymphoma-inducing (IARC, 1996, 1998), whereas oncogenic viruses have recently been suspected (Metayer *et al*, 1998).

We analysed the data for 78 894 patients registered with non-Hodgkin lymphoma as a first primary malignancy in England and Wales during the period 1986–1999, some 86% of those eligible. Nine per cent of cases were excluded from survival analysis because their recorded survival was zero (date of diagnosis same as date of death): most of these will have been registered from a death certificate only (DCO); hence, their date of diagnosis and their duration of survival were both unknown. These cases could not be reliably distinguished from cases with true zero survival in the national data. The proportion of cases excluded from analysis as DCO was similar in all deprivation groups, and this is unlikely to have had a material impact on estimates of trend or socioeconomic gradient in survival. A further 3% of patients were excluded because the lymphoma was not their first primary malignancy, along with 1.8% whose vital status was unknown at 5 November 2002, when the data were extracted for analysis.

## Survival trends

**Figure 1:**
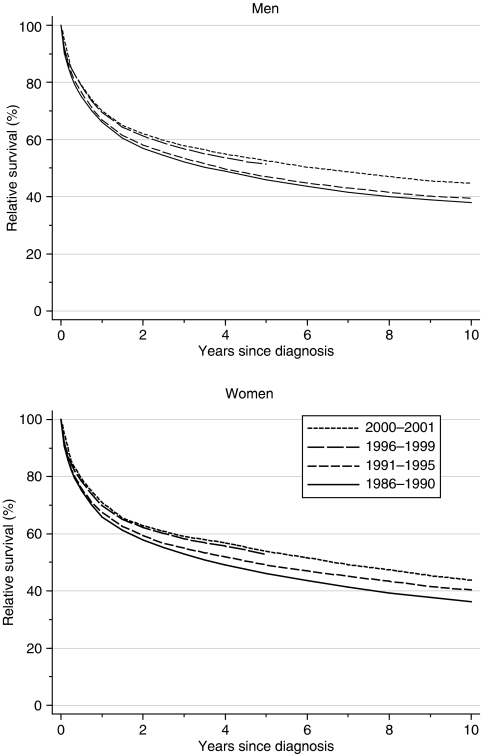
Relative survival (%) up to 10 years after diagnosis by sex and calendar period of diagnosis: England and Wales, adults (15--99 years) diagnosed during 1986--1999 and followed up to 2001. Survival estimated with cohort or complete approach (1986--1990, 1991--1995, 1996--1999) or hybrid approach (2000--2001) (see Rachet *et al*, 2008).

One-year survival rose from 66 to 70% in both sexes: after adjustment for differences among deprivation groups, that represents an increase of approximately 2% every 5 years (Table 1, Figure 1). Five-year survival rose more quickly than 1-year survival, from 46% for patients diagnosed during 1986–1990 to approximately 52% for those diagnosed during 1996–1999, a deprivation-adjusted increase of approximately 4% every 5 years. Both rates of increase are statistically significant. For patients diagnosed during 1996–1999, the 5-year relative survival reached 51% for men and 53% for women.

Hybrid analysis of the survival probabilities observed during 2000–2001 (Brenner and Rachet, 2004) suggests that the recent improvement in survival observed during the late 1990s is likely to continue (Figure 1, dashed line). Survival rates predicted for those diagnosed during 2000–2001 are approximately 70–71%, 53–54% and 44–45% at 1, 5 and 10 years after diagnosis, respectively (Table 1).

## Deprivation

**Figure 2:**
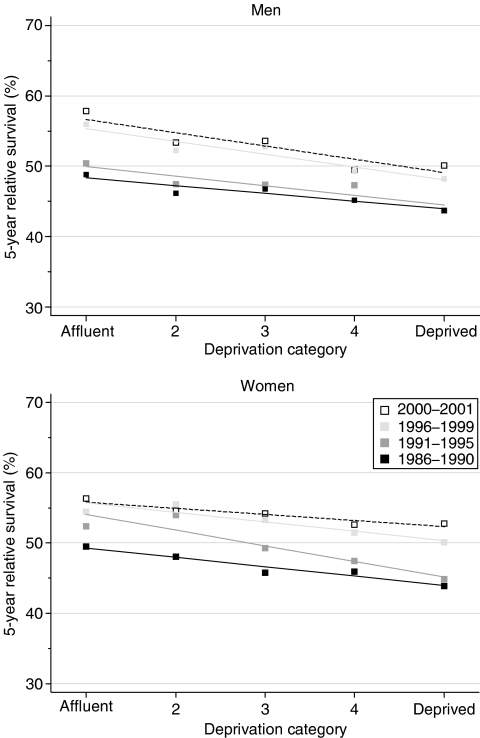
Trends in the deprivation gap in 5-year relative survival (%) by sex and calendar period of diagnosis: England and Wales, adults (15--99 years) diagnosed during 1986--1999 and followed up to 2001.

Survival at 1, 5 and 10 years after diagnosis is substantially and systematically higher among more affluent groups. Among more than 26 000 patients diagnosed during 1996–1999, the differences in 1- and 5-year survival between the most deprived and the most affluent groups were estimated at 5–7% (Table 2, Figure 2).

After adjusting for temporal trends in survival, the fitted deprivation gap is statistically significant in every case, with the exception of 10-year survival for patients diagnosed during 1986–1990.

The deprivation gap in survival has remained fairly stable: the slight widening (approximately −1% every 5 years) is not statistically significant (Table 2). The exception here is 10-year survival for women, where the deprivation gap has increased significantly.

Short-term predictions with hybrid analysis suggest that the socioeconomic differences in survival up to 10 years after diagnosis are likely to remain wide for the foreseeable future (Table 2).

## Comment

Steadily improving survival for patients with non-Hodgkin lymphoma in England and Wales during the 1990s continues a trend that has been evident for more than 20 years. Five-year survival for patients diagnosed during the early 1970s was approximately 30%, reaching 40–45% in the 1980s (Coleman *et al*, 1999) and now 52% for patients diagnosed in the late 1990s. This steady trend probably reflects progress in treatment, especially radiotherapy and chemotherapy, since the early 1980s. Survival in England and Wales is still lower than, although close to, figures reported from other western European countries (Sant *et al*, 2003).

The improvement in overall survival has not led to greater equity in outcome. Survival for the least affluent remains substantially lower than for the most affluent, even after adjustment for the differences and trends in background mortality among deprivation categories; without that adjustment, the observed difference in survival would be even greater.

## Figures and Tables

**Table 1:** Trends in relative survival (%) by sex, time since diagnosis and calendar period of diagnosis: England and Wales, adults (15–99 years) diagnosed during 1986–1999 and followed up to 2001

		**Calendar period of diagnosis^a^**									
		**1986–1990**		**1991–1995**		**1996–1999**		**Average change (%) every 5 years^b^**		**Prediction^c^ for patients diagnosed during 2000–2001**	
**Time since diagnosis**		**Survival (%)**	**95% CI**	**Survival (%)**	**95% CI**	**Survival (%)**	**95% CI**	**Survival (%)**	**95% CI**	**Survival (%)**	**95% CI**
1 year	Men	**66.0**	(65.1, 66.8)	**66.9**	(66.1, 67.6)	**69.5**	(68.7, 70.3)	2.2**	(0.6, 3.8)	**70.0**	(68.9, 71.2)
	Women	**65.8**	(64.9, 66.8)	**67.5**	(66.6, 68.3)	**69.9**	(69.0, 70.7)	2.4**	(0.7, 4.1)	**70.9**	(69.7, 72.1)
5 year	Men	**45.8**	(44.8, 46.9)	**47.0**	(46.1, 47.9)	**51.3**	(50.2, 52.4)	4.0**	(2.0, 5.9)	**52.6**	(51.2, 54.0)
	Women	**46.2**	(45.1, 47.2)	**49.1**	(48.1, 50.0)	**52.7**	(51.5, 53.9)	3.6**	(1.4, 5.8)	**53.9**	(52.4, 55.3)
10 year	Men	**38.0**	(36.9, 39.1)	**39.4**	(38.3, 40.5)			**3.5**	(−0.4, 7.3)	**44.7**	(43.1, 46.2)
	Women	**36.2**	(35.1, 37.3)	**40.3**	(39.1, 41.5)			8.7**	(4.5, 12.9)	**43.8**	(42.1, 45.4)

CI=confidence interval.

a Survival estimated with cohort or complete approach (see Rachet *et al*, 2008).

b Mean absolute change (%) in survival every 5 years, adjusted for deprivation (see Rachet *et al*, 2008).

c Survival estimated with hybrid approach (see Rachet *et al*, 2008).

^**^P<0.01.

**Table 2:** Trends in the deprivation gap in relative survival (%) by sex, time since diagnosis and calendar period of diagnosis: England and Wales, adults (15–99 years) diagnosed during 1986–1999 and followed up to 2001

		**Calendar period of diagnosis^a^**									
		**1986–1990**		**1991–1995**		**1996–1999**		**Average change (%) every 5 years^b^**		**Prediction^c^ for patients diagnosed during 2000–2001**	
**Time since diagnosis**		**Deprivation gap (%)**	**95% CI**	**Deprivation gap (%)**	**95% CI**	**Deprivation gap (%)**	**95% CI**	**Deprivation gap (%)**	**95% CI**	**Deprivation gap (%)**	**95% CI**
1 year	Men	−5.1**	(−7.6, −2.5)	−7.7**	(−10.0, −5.5)	−5.9**	(−8.3, −3.6)	**−0.4**	(−2.2, 1.4)	−6.7**	(−9.9, −3.4)
	Women	−5.0**	(−7.7, −2.2)	−8.0**	(−10.4, −5.6)	−5.7**	(−8.1, −3.2)	**−0.3**	(−2.3, 1.6)	−4.7**	(−8.1, −1.3)
5 years	Men	−4.4**	(−7.3, −1.5)	−5.5**	(−8.1, −2.9)	−7.3**	(−10.4, −4.1)	**−1.5**	(−3.8, 0.7)	−7.5**	(−11.5, −3.6)
	Women	−5.3**	(−8.4, −2.2)	−8.9**	(−11.6, −6.1)	−5.4**	(−8.9, −1.9)	**−0.4**	(−2.8, 2.1)	**−3.5**	(−7.6, 0.7)
10 years	Men	**−2.1**	(−5.2, 1.0)	−4.9**	(−7.9, −1.8)			**−2.7**	(−7.1, 1.6)	−7.0**	(−11.5, −2.5)
	Women	**−2.7**	(−6.0, 0.5)	−8.7**	(−12.2, −5.3)			−6.0*	(−10.7, −1.3)	−8.2**	(−12.9, −3.4)

CI=confidence interval.

a Survival estimated with cohort or complete approach (see Rachet *et al*, 2008).

b Mean absolute change (%) in the deprivation gap in survival every 5 years, adjusted for the underlying trend in survival (see Rachet *et al*, 2008).

c Survival estimated with hybrid approach (see Rachet *et al*, 2008).

^*^P<0.05; ^**^P<0.01.
